# The dairy chains in North Africa (Algeria, Morocco and Tunisia): from self sufficiency options to food dependency?

**DOI:** 10.1186/2193-1801-2-162

**Published:** 2013-04-16

**Authors:** Mohamed Taher Sraïri, Mohammed Tahar Benyoucef, Khemais Kraiem

**Affiliations:** Department of Animal Production & Biotechnology, Hassan II Agronomy and Veterinary Medicine Institute, P.O. Box 6 202, Madinate Al Irfane, 10 101 Rabat, Morocco; Department of Animal Productions, National High School of Agronomy, El Harrach, Algiers, Algeria; Department of Animal Resources, Food Technology and Rural Development, Higher Institute of Agricultural Science, Chott Mariam, Tunisia

**Keywords:** Cattle, Dairy chains, Fragmented offer, North Africa, Quality, Water productivity

## Abstract

The Maghreb countries (Algeria, Morocco and Tunisia) have experienced since the early 1950s a rapid demographic growth coupled to a significant rhythm of urbanization. This has led to a marked increase in the demand of dairy products. In order to secure the supply, specific policies have been implemented. They mainly consisted in the establishment of a dairy industry, based on the processing of either raw milk produced locally (in Morocco and Tunisia) or imported milk powder (in Algeria). These divergent options have had significant consequences on the whole organization of the dairy chains in these countries, from cattle rearing practices, to milk collection and processing. They have also implied differences in milk and its derivatives’ prices and levels of consumption. The paper draws a comparative analysis of milk chains within the three countries: a supply mainly based on imports in Algeria, whereas in Morocco and Tunisia, the demand is satisfied by a chain relying on locally produced cattle milk. The paper also emphasizes on the future challenges that will have to be addressed: a rising volatility of milk and other strategic inputs’ prices (feed, machinery, cattle, etc.) in global markets, an improvement in consumers’ awareness about milk quality, a further pressure on natural resources (mainly soils and water) to get more raw milk, in countries already suffering an acute water stress. The article also establishes recommendations about specific issues related to the development of the dairy chains in the context of North Africa. These are mainly linked to the fragmented offer induced by numerous smallholder farms, which implies obvious difficulties to assess the hygienic and the chemical quality of milk batches delivered daily. Moreover, this fragmented offer also means that specific support programs will have to be designed, as the vast majority of farms are not dairy specialized, expecting both milk and calf crop from their herds.

**Electronic supplementary material:**

The online version of this article (doi:10.1186/2193-1801-2-162) contains supplementary material, which is available to authorized users.

## Introduction

Located in the Northern fringes of Africa, the Maghreb countries (Algeria, Morocco and Tunisia) have a long tradition with dairy products’ consumption. In fact, their original dietary habits used to be based on cereals and vegetables, and on a limited intake of animal products, mainly *leben* (fermented milk), which all contributed to a low prevalence of obesity and cardiovascular diseases (Mehio Sibai et al. 2010). Moreover, according to the Berber and the Arabic traditions, milk has a symbolic value of life and fertility, as it is often used, with dates, in ceremonies to welcome guests (Benchelah and Maka 2008). The three countries have experienced since the Independence era (early 1960s) a rapid demographic growth coupled to changes in the diet. As a consequence, a massive surge in food demand has occurred (Lampietti et al. 2011), particularly in animal products (meat and milk), implying, as in many other developing countries the need for a “Livestock Revolution” (Delgado et al. 2003). Therefore, specific policies had to be implemented to secure the supply of food and create wealth and labor opportunities through the use of the natural resources: arable and range lands, animal wealth and irrigation water. In the specific field of animal products, dairying has been intensively supported since the colonial era, given its better metabolic efficiency in comparison to meat production (Vermorel and Coulon 1998). Several policies were therefore applied in each country, in relation to their resources availability and the weight of the rural areas in their domestic affairs. Schematically, Algeria with its prominent fossil energy exports, relied mainly on imports of milk powder to provide its citizens with subsidized dairy products (Henry 2004), becoming nowadays one of the most important dairy products’ importer worldwide. At the opposite, Morocco and Tunisia made different choices, based on encouraging locally produced raw milk. Such options have had important consequences on the whole organization of the dairy chains within the three countries. Given that these policies have been established for more than 40 years, it is now possible to draw a comparative diagnosis of the three dairy chains. In fact, many social, economic and environment challenges remain associated to cattle rearing in North Africa, as it is a key activity to ensure food security. It induces however marked impacts on the environment, given its important needs of water (Sraïri et al. 2009a) and its effects on groundwater pollution by nitrates (Laftouhi et al. 2003). Moreover, the current volatility in the prices of feed and food, and its effects on the imports’ bill put additional pressure on the actors of the dairy chains within the three countries, as their intrinsic water stress and the expected climate change already threaten the prevailing agricultural activities (Schilling et al. 2012). The current study aims to show the most recent trends and future prospects of the dairy chains in Algeria, Morocco and Tunisia. It also attempts to investigate the drivers for the development of milk production, consumption, and trade in the region, and the constraints that will have to be confronted.

### The context of dairy production in the Maghreb region

#### History

Livestock rearing is an ancient activity in the Maghreb region. In fact, this area is characterized by an original population of native cattle which are locally known as the Brown Atlas breed. There is strong evidence that this breed has many morphological similarities with the sub Saharan N’dama breed, as pastoral tribes used to cross the Sahara desert in attempts to find feed and water for their herds (Smith 1992). Another evidence of cattle herding activities in the region is the presence of rock art, disseminated in many parts of the Maghreb area, representing scenes of cows’ milking (Dunne et al. 2012).

Milk production has remained for many centuries practised by pastoral societies. It is the need to intensify cattle production, as a consequence of the demographic growth of the twentieth century, that has induced the emergence of fodder crops to feed cattle, at a time where livestock feeding was almost based only on range land resources, cereal crops’ by-products (straw, stubble and bran), in addition to fallow (Davis 2007). Recent history in the Maghreb region is also characterized by a rapid demographic expansion and by growing rhythms of urbanization. The official figures reveal that the population has more than doubled from 1970 to 2012: 13.7 to 37.1 millions in Algeria, 14.9 to 32.7 millions in Morocco and 5.1 to 10.7 millions in Tunisia (World Bank 2012). This trend of population growth is expected to continue until 2050, though these countries have entered a phase of demographic transition at different paces. Further, the overall population of the three countries is expected to reach around 105 million people in year 2050 (Population Reference Bureau 2010). Coupled to urbanization rates currently exceeding 60%, as they were inferior to 15% in the beginning of the 20th century, this has implied a marked evolution in the consumption habits, with more meals eaten alone, outside home. This trend has induced a surge in the demand of animal products with emerging risks of a food dependency for the region (Sraïri 2011).

#### Geography and natural resources

The Maghreb countries are mainly characterised by a typical Mediterranean climate, with a long summer period (from May to the end of September) of intense drought and excessive heat, followed by erratic rainfall from autumn to spring (October to April) (Lionello et al. 2006). Another factor which affects the Maghreb climate is the marine influence, as it decreases the amplitude of temperatures in areas near to the seashore: the Atlantic Ocean in Morocco, and the Mediterranean in Morocco, Algeria and Tunisia. The average annual rainfall is well below 300 mm in vast areas of the Maghreb countries, implying arid to semi arid climates (Patricola and Cook 2010). Therefore water stress constitutes the main limiting factor to agriculture. The hydrological water stress index which measures hundreds of people per one million cubic meters of available renewable water is respectively 29, 11 and 23 in Algeria, Morocco and Tunisia respectively (World Resources Institute 2008). It implies that at the regional level, Algeria and Tunisia face the highest level of water stress, while in Morocco water is less scarce. Such figures will certainly worsen with the expected demographic growth and climate change and they may threaten a harmonious human development (Rijsberman 2006). The relative better figure in Morocco is related to the Atlas mountains which have favoured the implementation of a policy of dams and large scale irrigation schemes named the “1 million irrigated hectares” (Faysse et al. 2010).

In addition to irrigation policies, the agricultural output in the Maghreb remains largely related to the level of annual rainfall in rain fed areas (Thomas 2008), with no possibilities of irrigation and which are nutrients’ shortages for ruminants prone. In fact, in the three countries, the availability of arable land per capita remains limited (respectively 0.24, 0.29 and 0.49 ha in Algeria, Morocco and Tunisia), and irrigation possibilities are reduced (Table 1). With regard to milk production, the contribution of the rain fed areas is highly variable and there is an ongoing trend towards localizing the dairy activity in the irrigated basins, where water availability enables to alleviate the effects of drought. For example, in Morocco, the irrigated regions which represent less than 15% of the total arable land contribute to up to 60% of the total annual output of milk (MAPM 2011).

**Table 1:** **Key statistics of the Maghreb countries**

	Algeria	Morocco	Tunisia
Country area (10^3^ ha)	238,174	71,085	16,215
Agricultural area (10^3^ ha)	8,459	9,232	5,045
Irrigated area (10^3^ ha)	907	1,454	345
Population in 2012 (× 1,000)	37,120	32,678	10,673
Urban population (%)	63	55	65
Life expectancy	72	74	75
GDP per capita - US $* (2009)	7,740	4,108	7,520
Contribution of agriculture to GDP (%)	8.3	17.1	10.6

* Purchasing power parity.

Source: CIA, 2010 and UNDP, 2009.

#### Animal resources

Another important characteristic of the dairy production in the Maghreb area is the reduced contribution of non cattle species to the overall output. In fact, because of the harsh environments which prevail in many parts of the region, small ruminants (sheep and goat) flocks are mainly used for meat production. The existing breeds of these species have not been selected for milk yield, at the exception of the Sicilo Sarde nucleus in Tunisia (Atti et al. 2006), where the vicinity with Sicily and the Ottoman colonization have induced an old tradition of cheese consumption made from ewe milk. Thus, the official figures reveal that milk from non cattle species (small ruminants and camel) represents respectively 21.3, 5.1 and 3.7% of the overall output in Algeria, Morocco and Tunisia (FAO STAT 2012), and its industrial processing remains rather weak. Therefore, integrated dairy chains rely mainly on cattle milk.

**Figure 1:**
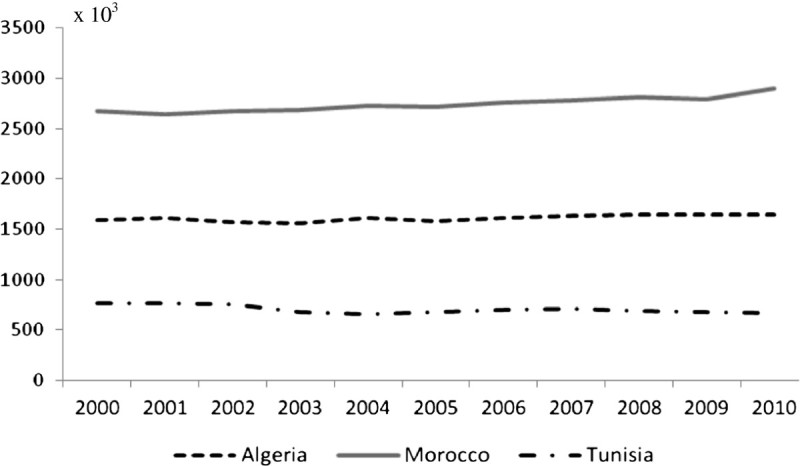
**Recent evolutions of cattle numbers in the Maghreb countries (2000–2010).**

**Figure 2:**
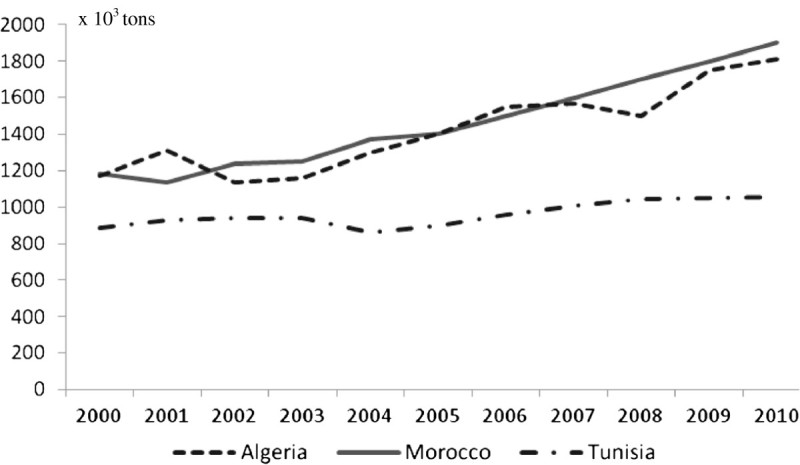
**Recent evolutions of cattle milk output in the Maghreb countries (2000–2010).**

Cattle numbers vary significantly among the Maghreb countries: respectively 1.6, 2.8 and 0.6 millions in Algeria, Morocco and Tunisia. These figures showed very slight evolutions in recent years (Figure 1), as the official policies in the three countries encourage an improvement of the average milk yield per cow rather than the increase in the number of cattle. To achieve such an increase of the milk output, one of the most prominent measures adopted was a program of crossbreeding of local strains with high genetic merit breeds like the Holstein, the Montbéliarde and the Brown Swiss. Consequently, imports of cattle of such breeds were encouraged. In Morocco, such imports are nowadays subsidized to support the constitution of a local population of such breeds. As a consequence, the imports of pregnant heifers have reached since the early 1960s respectively some 387,000, 350,000 and 80,000 in Algeria, Morocco and Tunisia. In addition to cattle imports, the Maghreb countries have all implemented programs of artificial insemination (AI), using semen of high genetic merit dairy cattle. The official figures reveal that the number of AI reached in year 2011 some 204,600; 320,000 and 305,000 in Algeria, Morocco and Tunisia. Though the number of AI is increasing steadily, its efficiency could be significantly improved as the conception rate in herds is frequently superior to 2 (Sraïri and Farit 2001). All together, these measures have implied a stable increase in the population of crossbred and purebred dairy cattle, at the expense of local breeds, which show a limited dairy potential (less than 1,000 kg per lactation). It is estimated that purebred dairy cows which used to represent less than 10% of the total cattle population in the early 1970s have increased to 17, 23 and 55%, respectively in Algeria, Morocco and Tunisia. The differences may be explained by the dairy policies adopted. In Algeria, milk powder imports have constituted a significant constraint for the local development of raw milk production and collection. They have also hindered the connexion between agricultural services and dairy cattle farms. Milk imports have also induced an insufficient interest in animal recording systems (Benyoucef and Abdelmoutaleb 2010). In Morocco, the constraints are related to the geography characteristics where an important population of local cattle is located in remote mountainous areas. These cattle breeding intervention measures coupled to continuous efforts to promote dairy intensification through irrigated forage, concentrates use and mechanic milking extension have prompted a continuous increase in the cattle milk output in the Maghreb countries (Figure 2).

### The dairy policies adopted and their consequences

Since the early 1960s, with the Independence era, the agricultural policies and the management of rural areas have played an important role in the development of the Maghreb countries. That is particularly true in Morocco and Tunisia, whereas in Algeria, the petroleum sector was more prominent. Therefore, in both Morocco and Tunisia, the major objective was to try to attain self-sufficiency in the most commonly consumed food products (cereal grains, milk, sugar, etc.), whereas in Algeria, the options were quite different, the authorities relying on gas exports to ensure the supply of the population with imported goods (Benyoucef 2005; Aït Amara 2009). In the particular field of the dairy chains, these options have had marked consequences.

#### Structure of milk production and collection

The main characteristics of cattle milk production in the Maghreb region is a fragmented offer, induced by numerous smallholder farms. The official figures indicate that in Algeria, Morocco and Tunisia, farms with less than 5 cows and which rely on less than 5 ha represent almost 80% of the total number of farms delivering milk to the supply chain. Such a structure implies a relatively high number of farms with cattle (respectively 212,000; 720,000 and 112,000 in Algeria, Morocco and Tunisia), with marked differences in their production characteristics: genetic type of cattle, feeding systems, respective weight of meat and milk in the production strategies, etc. Attempts to establish typologies of farms on their functioning characteristics have revealed that variables such as the efficiency of feed resources conversion to milk, the level of dairy intensification (mainly milk yield per lactating cow) and the economic profitability are the most decisive, with no effect of farms geographic location (whether in an irrigated scheme or a rain fed suburban region) (Sraïri et al. 2009b). Therefore, cattle’s feeding represents an important constraint in this region and has significant consequences on farms’ performances. Thus, the climate characteristics of the Maghreb region induce marked effects for the sustainability of intensive dairy farming, because of frequent drought seasons, which also affect the irrigated areas (Sraïri and Ilham 2001), as water volumes within dams is not sufficient to support an important demand from various crops, particularly in summer time, where temperatures might reach 50°C. The main constraint is represented by frequent insufficient and imbalanced dietary rations, which alter milk yield. Many research studies have demonstrated that the average annual milk yield per cow in conventional herds, even with high genetic merit dairy breeds or crossbred cows, does not exceed 2,500 to 3,000 kg (Madani and Mouffok 2008; Rekhis et al. 2007; Sraïri et al. 2009b), whereas in Tunisia, in herds with purebred dairy cattle breeds it reaches 3,500 to 4,000 kg (GIV Lait 2012). At farm level, dairy cattle feeding systems in various areas of the Maghreb region suffer from acute limitations, as cattle load (number of animals per ha of fodder) is frequently too high. Moreover, as the vast majority of cattle farms are not dairy specialized, there is often a significant competition for feed resources between lactating cows and growing calves, which means that farmers have to make painful choices between the two functions. To overcome such limitations, farmers used to rely on two options: *i*) the important use of off-farm feed resources, through purchased feed like cereal grains (maize and barley mainly), crop by-products, such as beet pulp and bran, and proteaginous meals (soya been and sunflower) which may represent up to 60% of the total energy intake (Kadi and Djellal 2009; Sraïri and Kessab 1998), and *ii*) the incorporation of lignified poor roughages at high levels in dietary rations, given that these resources like straw or hay can easily be harvested and transported from cereal specialized regions to areas with an intensified cattle activity (Annicchiarico et al. 2005). Those two options show obvious limitations. First of all, in the current context, given the soaring prices of feed in global markets and the stagnating farm gate milk prices, the use of off-farm resources may hamper significantly the economic profitability of the dairy activity (Rejeb Gharbi et al. 2007). Second, the use of cereal straws and poor roughages (lignified oat hay) as the pillar of cattle feeding systems in dry seasons may also decrease significantly milk yield. In fact, such feeding resources show a reduced nitrogen content (less than 10%) and a limited digestibility (less than 50%) which cannot sustain the feeding requirements of high genetic merit cows (Ørskov 1999). As a consequence, milk yield in dry periods often decreases dramatically, as farmers are not well aware about the techniques of dietary rations complementation and they even often ignore cattle requirements exactly. Moreover, such feeding systems characteristics also imply frequent reproduction failures, which contribute to alter the overall farms’ profitability (Sraïri and El Khattabi 2001; Madani et al. 2002). To enhance the performances, support programs for farmers, based on an effective follow-up of herds, are urgently required.

Raw milk fragmented offer in the Maghreb countries also implies that a collection infrastructure has to be established. It often consists in dairy collection co-operatives, which are generally a group of neighbouring farmers who install a refrigeration device to collect milk and aggregate it before delivering their output to dairy processing units. The evolutions and the performances of this collection infrastructure in the Maghreb countries are rather contrasted. In fact, with a dairy policy mainly relying on milk powder imports to secure the supply since the early 1970s, the development of an efficient chain using local raw milk has not been achieved yet in Algeria. The unfair competition with cheaper imported milk powder has not enabled local operators to invest in the collection network (Benyoucef, 2005). However, with the global food crisis of 2007 and its effects on milk imports’ bill, the Algerian authorities have effectively activated the National Inter professional Milk Office (ONIL), as a regulation agency with the mission to organize the dairy sector. One of its main objective was to increase significantly the rate of raw milk collection, as it reached 22.4% of the national output in 2011, while this figure did not exceed an average value of 13%, from 2000 to 2010 (DRDPA 2011). The relative improvement of milk collection rate has been made effective through a regular increase in the financial incentive given to collection centres, from 0.03 to 0.06 and finally 0.09 US $ per kg of milk, respectively in 2000, 2003 and 2009. As a consequence, in year 2011, it is estimated that raw milk collection has reached the 560 million litres (DRDPA 2011). These evolutions still mean however that almost 80% of the milk output escapes from industrial processing, being consumed on-farm or sold in short scale local circuits. As an example, in some peripheral regions of the south of Algeria, the dairy chains remain embryonic, because of the absence of industrial processing units. Milk produced by dairy cattle farms outside the oases is sold directly to consumers. By contrast, milk from small ruminants’ species (goats and ewes) is consumed on-farm, by the breeders’ family members (Benyoucef and Boubekeur 2010).

In Morocco and Tunisia, the situation is rather different. Milk collection centres play a crucial role within the dairy supply chain. They also constitute vital operators in regional political affairs, given their interactions with local communities and their steady incomes. The number of milk collection points has been continuously increasing in both countries as it reached in year 2011, 230 in Tunisia and more than 1,450 in Morocco. They gather up to 60% of the total output in the two countries (Table 2).

**Table 2:** **Main indicators of the dairy chains in the maghreb countries (2010)**

	Algeria	Morocco	Tunisia
Number of cattle (x 1,000)	1,600	2,800	600
Pure breed dairy cattle (%)	17	23	55
Cattle milk output (× 10^3^ tons)	1,811	1,900	1,059
Non cattle milk in the overall milk output (%)	21.3	5.1	3.7
Formal milk collection rate (%)	14	65	63
Number of industrial milk processing units	144	44	41
Average annual milk consumption (kg per capita)	100	55	110
Self sufficiency rate in dairy products (%)	49	89	95

Source: FAO STAT, 2012 and national statistics compiled by the authors.

Raw milk collection centres’ performances are generally acceptable, as their main function is to gather milk from farmers and to sell it to industrial plants. In Morocco, their gross margins are secure, as there is a differential between farm gate price which is paid to farmers and the price of milk sales to industrial processors. Generally, that differential is up to 5 to 10% of farm gate milk price, whether farmers deliver by their own means their daily production or the collection centre sends a small pick up to farms to get milk.

In Tunisia the State authorities implemented since 1988 a subsidy for milk collection centres which reached 0.025 US $ per kg in 1994 to secure a reasonable margin. Until now, the organisation of the dairy chains in both Morocco and Tunisia show that milk collection points have been successful, as they allow farmers even in remote areas to integer industrial food chains and earn a vital daily income. However, such structures show evident limitations when it comes to assessing regularly individual milk quality and rewarding it consequently (Le Gal et al. 2009).

#### Dairy processing operators in the Maghreb countries

The operators in the industrial dairy processing sector in the Maghreb area can be classified according to three criteria: their industrial capacity (quantity of milk processed yearly), their status (totally private, co-operative, or State owned) and the type of products they sell (drink milk and/or dairy derivatives). In addition to these industrial processors, there are also numerous artisanal dairy units.

In Algeria, the leading public company is GIPLAIT, an old state group which manages some 14 units disseminated throughout the country (Bencharif 2001). It is currently undergoing restructuring with the aim to privatise some of its plants. They mainly operate with imported milk powder and milk fat. They supply the market with almost 70% of the total quantities of subsidized drink milk and 30% of the dairy derivatives, mainly yogurts and cheeses (Benyoucef 2005).

In addition to these State run units, there are 130 private units of various industrial capacities in the dairy processing sector. The vast majority of these factories (99) operate with imported milk powder to produce rather drink milk or dairy derivatives. Given the recent changes in the dairy policy in Algeria, and the emergence of a strong political will to encourage locally produced raw milk channels, some processing units have settled recently with the aim of avoiding the use of imported milk powder. There are nowadays 21 units of various capacities using raw milk to get drink milk (Ultra High Temperature - UHT) and 10 processing units which use local raw milk to produce dairy derivatives. These recent developments have also been encouraged by the intervention in the dairy processing activity of international groups like “Sodiaal” (brand name Candia) and “Danone”, who are seeking joint ventures with local groups with the objective to invest in this rapidly growing supply chain, in a country recently integrating the market economy (Cheriet et al. 2008).

In Morocco, the dairy processing sector is rather different, as from its very beginning it has always been totally private. It consists in some 44 societies which may be classified in four different types.

The first one gathers private industrial operators run by independent societies. There are four main societies operating in this sector, of which the “Centrale Laitière” group, occupying a leading position with almost 55% of the volumes processed. This society has established strong ties with the international dairy group “Danone”, which controls in 2012 some 67% of its capital. Its joint venture with “Danone” provides “Centrale Laitière” with two important assets: technical know-how and the ability to sell products under the well-known “Danone” label (Jazi 2003). In 2008, following the global food crisis, “Centrale Laitière” invested massively in a milk dryer to ensure domestic supply. This facility is currently the most important milk dryer in Africa. In fact, this has been a logical step for the company to avoid milk losses in times of excess supply (especially during rainy seasons in years with important precipitation). Therefore, the milk is dried and conserved until periods of milk shortage, as this also allows the company to decrease its imports of milk powder, which at the time was very expensive. Similarly, “Centrale Laitière” has launched in year 2008, following the food global crisis an important project of dairy cattle production called “Lait Plus”. This intensive dairy farm aims to rear some 10,000 lactating cows in the Gharb irrigated scheme, also characterized by its average annual rainfall above 600 mm. This was an additional step adopted by the “Centrale Laitière” group to secure its supply of raw milk and to try to improve the quality of the raw matter it processes. Such a project is also supported by the ongoing trend of agricultural intensification promoted by the Moroccan authorities, known as the “Green Morocco Plan” (MAPM 2011).

In addition to “Centrale Laitière”, there has been in 2005 the emergence of another important player in the private sector of dairy processors. This is the “Safilait” company, known locally by its brand “Aljibal” (Mountains in Arabic). The plant is situated in the irrigated scheme of Tadla (centre East of Morocco) where the company runs a dairy farm - Agroplus - of more than 3,000 cows of both Holstein and Montbéliarde breeds. The annual processed quantity of milk reaches some 30,000 tons representing around 4% of the national milk production. During the last 5 years, the “Aljibal” brand name has gained a reputation of good quality and its products can be found in supermarkets in large cities.

Apart from these two operators, there are also two other private smaller groups. The first one is the “Domaines Agricoles”, a private company mainly retained by the Royal family. It is producing niche market dairy products under the brand “Chergui”. This operator processes only milk from three of its own farms located in the North of Morocco, with a herd estimated to contain some 1,000 lactating cows. Its dairy plant processes around 9,000 tons annually, exclusively in milk derivatives, such as yogurts and *leben*, sold about 10% more expensive than the average price of conventional products of other brands.

Finally, there is also the global group “Nestlé”, present in Morocco but only with some high value products such as ice creams, dairy specialities for infants and milk powder. Its dairy basin is located in the Doukkala large scale irrigation scheme where it processes around 3% of the volumes collected by industrial plants.

The second type of dairy processors is represented by a large national cooperative, called “COPAG”, which is based in the Southern region of Souss-Massa. Over the past twenty years, this cooperative has become a strong competitor to “Centrale Laitière”. “Copag” incorporates some 11,000 dairy farmers milking around 40,000 cows. The co-operative represents some 20% of the total milk processed and its market shares are progressively increasing, as the quality of its drink milk and dairy preparations, sold under the brand name “Jaouda” (Quality in Arabic) has been recognized. Moreover, its co-operative status lets the company benefit from certain goodwill among Moroccan consumers (Errahj et al. 2009).

Other than the large players, there is a host of smaller processing co-operatives based in a number of different dairy basins supplying only their immediate geographical markets. This part of the processing sector is very complex and highly fragmented, as it gathers some 30 co-operatives representing less than 15% of the milk volumes processed. Some of these small co-operatives were dairy processing pioneers in Morocco (“Le Bon Lait” in the Marrakech region, “Colait Extralait” in the Gharb, etc.) but they are currently struggling to survive as they face severe financial and technical problems such as increases in milk prices and other strategic inputs’ costs (such as imported milk powder, butter, energy, etc.). They produce only a narrow range of dairy products that is not usually stocked by supermarkets and they only have little know-how when it comes to milk processing. Some of them, in an attempt of resilience, like “Le Bon Lait” are currently establishing a joint venture with the French leading dairy co-operative group “Sodiaal” to market the brands Candia and Yoplait in the Moroccan market.

Finally, the traditional processing sector also deserves mention. These small operators are known locally as *mahlabates*. They are mainly located in popular suburbs in large cities where they only serve a small niche market. There are however no official data specifying the exact milk volumes going through this informal sector, though it is estimated they process around 10 to 15% of Morocco’s raw milk. These circuits benefit from both farmers’ and consumers’ acceptance. Indeed, farmers manage to sell their milk at prices 15 to 20% higher than to collection centres.

Similarly to Morocco and Algeria, the milk processing sector in Tunisia is fragmented, with many companies of different size and ownership structure. Dairy processing activities are practiced by 41 societies: 8 which produce mainly drink milk (Ultra High Temperature), 4 are specialized in yogurts, 28 manufacture cheese and 1 which processes milk to powder.

The “Delice Danone” group is a strong leader in the dairy processing activities in Tunisia. It has two different plants, one for drinking milk and the other for yogurts’ production. This group represents almost 50% of the overall drink milk market shares and 70% of total sales of yogurts (Ben Mahmoud and Jemni 2008). Two other important dairy processors are Vitalait and Laino, both active in drink milk and yogurts, but with limited market shares. Tunisia has a long tradition of cheese processing and some companies are specialized in that activity. This is the case of Centrale des Produits Laitiers Souani, which represent 60% of total cheese production of the country.

**Figure 3:**
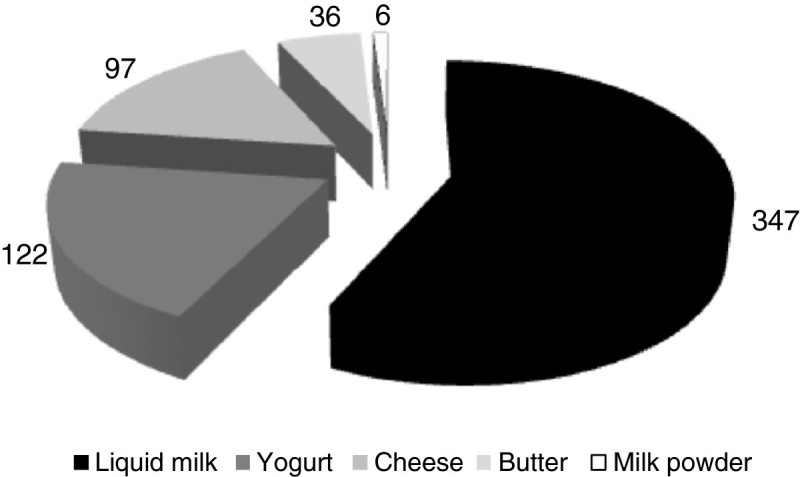
**Split of the Tunisian dairy sector by product (average annual output -10** ^**3**^ **tons-, 2000–2009).**

Drink milk represents more than 75% of total milk processed, whereas yogurts and cheeses represent only 13 and 8% of total volumes (Figure 3). The 4% remaining are converted to dried milk, particularly in periods of high production, used in case of milk shortage.

In fact, in Morocco, as the supply is mainly based on the deliveries of local raw milk, the seasonality of production constitutes a real constraint to dairy processors. As periods of milk excess are frequent in the rainy season, it creates significant tensions to remunerate farmers. Some dairy processors have tried to overcome such problems by drying milk and using it in periods of limited production. In other situations, farmers may not be paid regularly, adding pressure on the profitability of cattle rearing (Sraïri and Chohin Kuper 2007). In Tunisia, two distinct periods of production are observed. The period of high output starts in spring and ends in the middle of summer. It is characterized by frequent milk refusal by processing units, because of its low quality (low density, high acidity, high plate counts, etc.). At the opposite, during the period of low production (from the end of summer until the end of winter), due to low forage availability, all the milk production is accepted by the processing units, regardless of its quality, because of the lack of raw matter.

#### Milk prices and their effects on milk and dairy derivatives’ consumption

Milk prices and their variations according to quality criteria are crucial indicators of the dairy chains’ governance (Valeeva et al. 2007). In the Maghreb countries, there are contrasted policies which have been implemented by the authorities, with regard to milk prices. In Algeria, given the aim to secure the supply mainly with imported commodities, the structure of the dairy products’ market is linked primarily to the subsidized drink milk reconstituted from imported powder. This good represents up to 52% of the dairy products’ overall consumption. Its price has been fixed at 25 DA per litre (0.3 US $). Such a subsidized product has been harmful to the integration of local raw milk in the supply chain, as it is estimated that it represents less than 10% of the dairy uses in the country. However, because of the soaring prices of imported milk powder and other dairy products during the global food crisis of 2007, a significant trend to increase the use of local raw milk has occurred. This has been noticed through the adoption by the State authorities in year 2008 of a series of incentives to increase raw milk production (0.14 US $ per kg), collection (0.06 US $ per kg) and industrial processing (0.07 US $ per kg) (DRDPA 2011). In addition, State authorities have adopted other incentives to promote cattle rearing (19 US $ per conception through AI, a subvention of 30% of the investments in milking machine, ensiling material, etc.) and milk collection (up to 30% of the investments in cooling devices). All these intervention measures aim to decrease the bill of dairy imports, and try to promote a sustainable supply chain based on locally produced fresh milk. As a consequence, farm gate milk price which stagnated for many years at 0.23 US $ per kg has increased in 2009 to 0.33 US $. During the same period, the price of the subsidized drink milk reconstituted from imported powder has remained administrated by the State to 0.27 US $. Given the lower price of the subsidized milk in comparison to raw milk, even before its processing, the Algerian authorities try to control the market of subsidized milk through ONIL. All together, the structure of the Algerian dairy market proves that the authorities prefer to promote mass consumption at the expense of the development of the local cattle industry. As a result, the average annual milk and dairy products’ consumption reaches almost 100 kg *per capita*. Milk powder accounts for 51.5 kg per capita, followed by drink milk (37.0 kg), fermented milk (8.7 kg), cheese (1,6 kg) and finally yogurt (1,2 kg) (ONS 2010).

In Morocco, milk prices are rather different. With the structural adjustment policies applied to the Moroccan economy at the beginning of the 1980s, the liberalization of the dairy chain’s governance has induced the end of State intervention. Therefore, from 1992, milk prices, whether at farm gate or at the consumer are totally free of any State intervention (Aït El Mekki and Tyner 2004). As the imports of dairy products are heavily taxed, the Moroccan market is mainly supplied with locally produced raw milk. Long term evolutions of farm gate and consumption milk prices reveal a significant trend of increase of the value perceived by dairy processors at the expense of cattle farmers (Sraïri and Chohin Kuper, 2007) (Table 3). Such an evolution has recently induced the increase of tensions within the dairy chain between farmers and dairy processors, particularly with the marked increase of major inputs’ prices (above all feed). In comparison to the Algerian situation, milk price at consumption remains higher (almost 0.75 US $ per kg of drink milk), as there are no subvention specific to that product. As a result, annual milk and dairy products average consumption is lower, as it is estimated to reach in 2010 some 55 kg per capita. Studies on the structure of milk consumption show that the consumers’ perception of dairy products is rather good, as they represent almost 8% of the overall food expenditures. However milk consumption suffers from the limited income of many households, particularly in rural areas (HCP, 2001). Therefore, important disparities in milk and dairy products’ consumption levels remain among consumers, according to their social background and the households’ income (Sraïri and Karbab, 2010).

In Tunisia, milk prices have been intensively linked to the intervention of the authorities. Rejeb Gharbi et al. (2007) have in fact clearly demonstrated that without border protection, dairy production is not profitable in Tunisia even under the best favorable hypothesis about water and concentrate feed prices. Therefore, farm gate milk price has steadily increased, to ensure the sustainability of cattle farmers. In year 2007, farm gate milk price reached a value of 0.36 US $ per kg, after an increase of almost 21% as a result of several protests of farmers due to the soaring prices of inputs. In addition to the State intervention through the increase of farm fate milk price, an incentive to encourage milk collection and processing was also implemented. It reached 0.025 US $ per kg of raw milk destined to milk collection centres (GIV Lait 2012).

Milk and dairy products’ consumption is estimated to reach 110 kg per capita in year 2011, and it has been steadily increasing since the 1980s, when it did not exceed 85 kg. In addition, the structure of dairy products’ consumption has evolved significantly, as drink milk quantities almost stagnated, whereas cheese and butter figures experienced marked increases (respectively 600 and 57% from the 1990s levels) (Dhehibi and Gil 2003).

**Table 3:** **Evolution of farm gate milk price and consumption milk price in Morocco - US $ (1970/2010)**

Year	Farm gate milk price (1)	Milk price at consumption (2)	(1)/(2) (%)
1970	0.06	0.12	51.4
1975	0.10	0.13	75.4
1980	0.16	0.23	68.6
1985	0.23	0.34	66.1
1990	0.29	0.45	63.9
1995	0.33	0.55	58.8
2000	0.33	0.60	54.4
2005	0.33	0.69	47.4
2010	0.33	0.71	46.9

Adapted from Sraïri and Chohin Kuper, 2007.

#### Milk and dairy products’ trade

**Figure 4:**
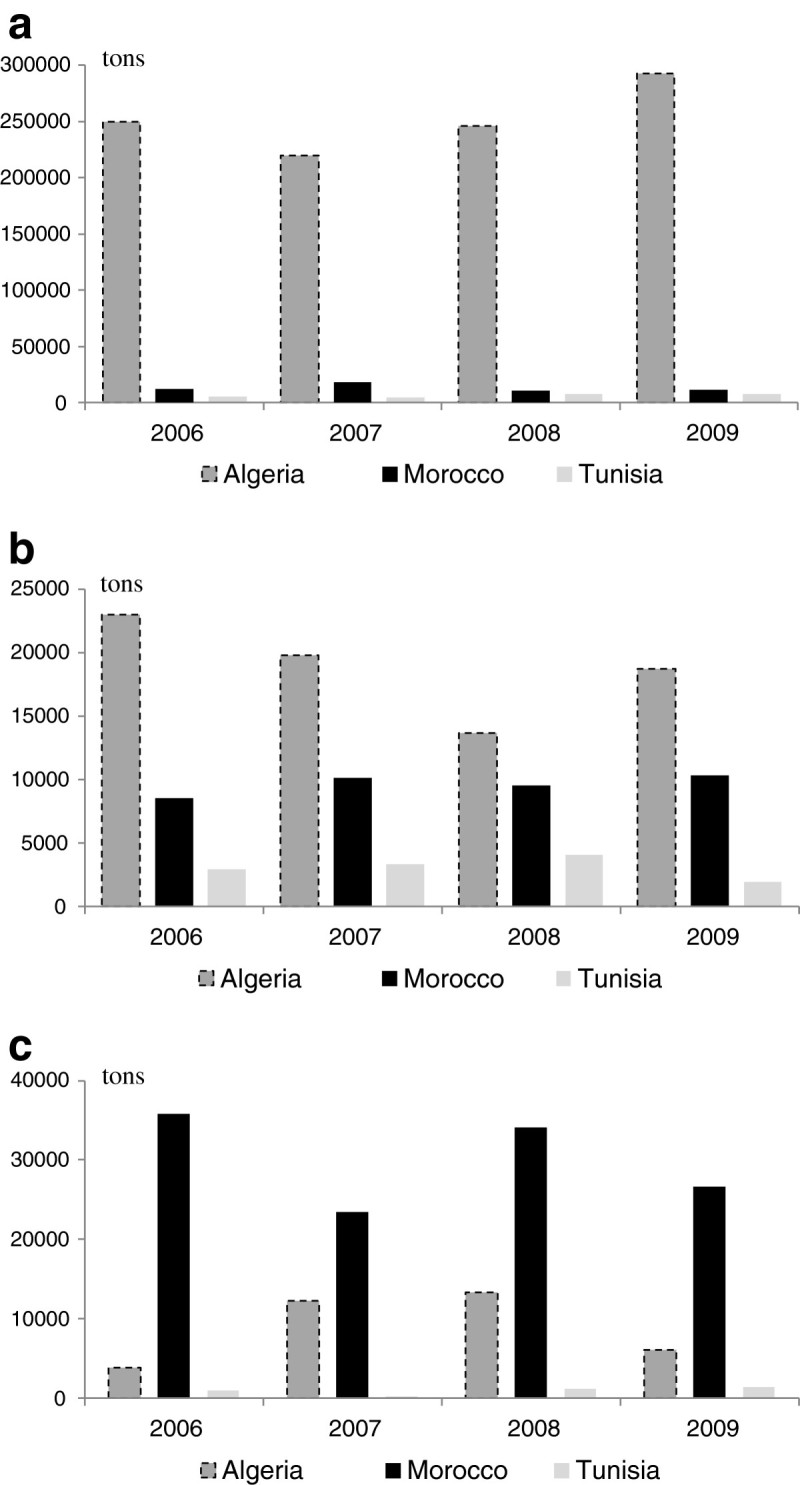
**Dairy products imports into the Maghreb countries, 2006–2009. a** Milk powder (both skimmed or whole milk dried) imports. **b** Cheese imports. **c** Butter imports.

The Maghreb countries are net importers of dairy products. In fact, the Algerian total dairy imports were valued at 1.2 billion US $ in 2008 (an average annual expenditure of 36 US $ *per capita*), and this value decreased to 859 million US $ in 2009, as a result of lower international commodity prices. This still accounted for 15% of the country’s all food imports, second only to cereal grains. The Algerian government controls the price for drink milk reconstituted from imported milk powder delivered only by ONIL. Algeria’s tariffs and duties on dairy products range from 5 to 47%. Proximity and good freight rates from Europe have always made trade with the European Union more advantageous and easier than for other countries. The structure of the Algerian dairy products is by far dominated by milk powder (whether skimmed or whole milk dried) which almost accounts for an annual volume of 252,000 metric tons (average value from 2006 to 2009) (Figure 4a). It is followed by cheese (an average of 18,900 tons per year, from 2006 to 2009), and finally butter (an average of 8,840 tons annually during the same period).

The Moroccan dairy imports are rather different. The overall value of dairy products imports reached 227 million US $ in 2010 (Office des Changes 2012). That corresponds for year 2010 to an expenditure of less than 8 US $per capita. Morocco’s tariffs/duties on dairy products range from 17.5% (cheese) to 32.5% (butter) and reaches 102% for milk powder and yogurts, in a strong sign that the authorities protect the local market, with high duties levied on dairy products.

As the domestic market is mainly supplied by locally produced raw milk, the Moroccan dairy imports are dominated by high value derivatives. Butter ranked first during year 2009 (26,600 tons) (Figure 4b), followed by milk powder and cheese, with almost the same volumes (respectively 11,500 and 10,300 tons) (Office des Changes 2012). The official data reveal that the main suppliers of dairy products to Morocco have varied significantly in recent years. Since year 2007, there has been a surge in US imports of dairy products, as a consequence of the free trade agreement signed between the two countries in 2004. Other traditional important suppliers of dairy products to Morocco are France for milk powder (9,400 tons), New Zealand and Ireland for cheese (respectively 2,300 and 2,000 tons) and New Zealand and Australia for butter (7,800 and 5,400 tons).

The Tunisian dairy imports are the most limited in the region. They did not exceed 34.3 million US $ in 2009 (3.2 US *$ per capita*). Tunisia’s tariffs/duties on dairy products range from 31 to 67%: yogurt and cheese (all types) duties - 67%, crude milk, milk powder and butter duties - 57% and whey duties - 31% (Tunisian customs, 2012). Milk powder is the most imported dairy good (7,780 tons in 2009), followed by cheese (1,920 tons) and finally butter (1,400 tons) (Figure 4c).

Finally, it is worth mentioning that the Maghreb countries do not export dairy products. Some of the huge quantities of milk powder imported by Algeria are however smuggled to neighbouring Morocco, Tunisia and Libya, but the exact quantities remain unknown. In addition, Tunisia has tried to export some drink milk to Algeria, particularly in period with raw milk excess (rainy seasons). However, given the seasonality of such exports, they cannot sustain all year long the demand of the Algerian market.

### Current challenges and future outlook

Many challenges lie ahead for the dairy chains in the Maghreb area if they want to achieve a sustainable development and contribute efficiently to the supply of the population. One of the most important is linked to the steady increase of milk volumes in a context of scarce resources, particularly water. In fact, given the climate constraints, no dairy intensification may be possible without the production of high quality irrigated fodder. The existing figures show that on farm water productivity through cattle can be significantly improved as, from irrigation practices to fodder yield and its conversion to cattle products (milk and live weight gain), there are numerous losses. In the specific case of the Tadla large scale irrigation scheme in Morocco, with a dairy chain relying on perennial alfalfa as the main fodder in numerous smallholder farms with herds of dual purpose (milk and meat simultaneously), it appeared that almost 1.8 and 10.6 cubic meters of water were necessary to get a single kg of milk and of live weight gain respectively (Sraïri et al. 2009a). Such results imply the use of huge amounts of water to ensure higher milk outputs. Unfortunately, many areas of the Maghreb countries are characterized by structural aridity and erratic rainfall which have already implied an unsustainable use of groundwater resources to intensify their agricultural activities (Wada et al. 2012).

Another important challenge for the Maghreb dairy chains is related to the complex issue of milk quality assessment and remuneration. In fact, in the intermediate collection structures, which gather milk deliveries from farmers before supplying the industrial process units, the quality of individual batches can hardly be analyzed due to technical, economic and logistic limitations. In the vast majority of situations, milk payment to farmers is only based on quantities delivered, although some collection structures try to avoid frauds by testing milk density or acidity (Le Gal et al. 2009). However, these tests do not provide any indication about milk chemical (fats and proteins) or hygienic (level of microbial contamination) quality. This implies that for the majority of smallholder farms there is no direct incentive to improve milk quality, as it won’t be rewarded.

The existing references on milk quality in the Maghreb region indicate that fat and protein contents may be acceptable, unless there are conditions of inadequate feeding: in farms relying mainly on concentrates with limited amount of forage, fat contents are generally low (Sraïri et al. 2005). But the most evident characteristics of raw milk in the Maghreb cattle systems is its poor hygienic quality, as its microbial load is generally 100 fold more important than international standards. This is due to poor hygiene at farm level (Aggad et al. 2009; Sraïri et al. 2009c).

As the current context of milk production is characterized by soaring prices of feed and stagnating farm gate milk prices, many farmers argue that milk quality could be a significant issue to improve their margins. Therefore, all the stakeholders within the dairy chains, from farmers to collection co-operatives and dairy processing units are requested to try to tackle that collective challenge. To upgrade the quality of raw milk in the Maghreb region, there is an urgent need to implement transparent milk payment systems. That has to be coupled to regular negotiations between the stakeholders on value chain distribution, and the way to increase it, particularly by promoting milk yield and quality.

This question raises another challenge for the dairy chains in the Maghreb area, which is in relation to the implementation of support programs destined to farmers. In fact, with the huge numbers of non specialized smallholder cattle farms, the feasibility of on farm follow-up and intervention is quite limited. The traditional State technical services withdrawal from the monitoring of farms has even worsened the situation, like in many other developing countries (Kidd et al. 2000). New channels of farmers learning and innovation are progressively emerging, like the collection co-operatives, but it may take time until they efficiently contribute to assist all the farmers (Faysse et al. 2012). As many shortfalls still characterize dairy farming, from insufficient and imbalanced dietary rations for milking cows to inadequate hygienic practices, on-farm support programs can be practical tools to improve milk yield and increase farmers’ income. Therefore, there is an urgent need to place support programs targeting dairy smallholder farms on the forefront of the agricultural policy agenda, if the expected increases in the milk output are effectively sought. Such programs should give a top priority to a continuous assessment of the nutrients’ content in the dietary rations used by farmers and try to balance that content with the effective requirements of milking cows. To achieve this aim, and given that both rations and nutrients’ requirements of cows are variable throughout the year, a proximity monitoring of farms is necessary (Sraïri et al. 2011).

In fact, all the Maghreb countries have planned a steady increase in their milk output in the near future. For the specific case of Morocco, an overall agricultural strategy, named the “Green Morocco Plan” has been implemented in 2008, and it projects the raw milk output to reach 5 million tons by year 2020 (MAPM 2008). Such a figure implies more than the double of the current milk production. The “Green Morocco Plan” is mainly based on optimistic hypotheses, where important dairy projects based on farms with more than 500 cows may be settled in the most favorable areas of the country, i.e. large scale hydraulic schemes and rain fed regions with more than 600 mm of average rainfall. However, the “Green Morocco Plan” almost does not mention the necessary increase in fodder quantities that would be needed to get such an increase in milk productivity. That constitutes another significant challenge for the dairy chains in the Maghreb countries, as the “Forage Revolution” is quite inexistent and heavy imports of feed being planned to improve animal production (Abdelguerfi and Ameziane, 2011).

Another significant challenge for the Maghreb dairy chains is in relation to the upgrading of the dairy products’ offer and consumption. This is linked primarily to the evolution of households’ incomes. Currently, the average consumption of dairy products remains limited and mainly dominated by drink milk. If it is to diversify towards more dairy derivatives with higher value, such as cheese, yogurts and light products, incomes will have to be improved, particularly for the vast majority of households with revenues not exceeding 3,000 US $ annually. Moreover, it will also mean that the local dairy processing units may get the necessary know-how to improve their products. That may also be prompted by the ongoing emergence of multinational dairy brands within the Maghreb markets, through joint-ventures with local units. In fact, in Algeria, Morocco and Tunisia, global dairy operators like “Danone”, “Nestlé” and “Sodiaal” are beginning to invest and their brand names are becoming common. Such a trend might also assist the overall upgrading of the local dairy chains, given the international standards brought by these operators with regard to milk quality and processing.

Last but not least, the free trade agreements being adopted by the countries of the Maghreb region with the EU and the US may also bear significant consequences for their dairy chains. They may induce the progressive decrease of the high duties levied on the dairy products, particularly in Morocco and Tunisia. That would also mean the end of the protection of the local market, with an acute competition from imported goods. Free trade agreements imply a vast array of measures which need to complement the agricultural reform efforts and preserve the economic and social stability in a sector of the economy which almost employ half of the population.

## Conclusion

The dairy chains in the Maghreb countries represent important players in the global dairy market, particularly because of the leading position of the Algerian imports of milk powder. These dairy chains will continue to grow, as a response to the demographic expansion and the expected improvement of the *per capita* Gross Domestic Product (GDP), which will certainly induce a diversification in the dairy products’ consumption towards more high value derivatives (yogurts, cheese and butter). To achieve such a growth, more raw milk will be needed, which implies the implementation of sustainable dairy cattle systems. Therefore, additional efforts will be needed in cattle farms to improve the water productivity through milk and meat, as the limited water resources certainly constitute the most important threat to the increase of milk output. This is particularly true in irrigated schemes located in arid regions, where non sustainable rhythms of use of groundwater have already induced the collapse of dairy farming and its delocalization in areas with more rainfall. On another hand, sound on-farm support programs targeting in priority smallholder units will be urgently needed, as they will surely remain the main actors in dairy farming. Such programs should aim to promote the overall farm performances, from irrigation efficiency, to fodder biomass yield and its conversion to milk and meat, given that such farms are rarely dairy specialized. In addition, more efforts to promote equity and a fair distribution of incomes throughout the dairy chains will be needed. This will have a direct link with the implementation of good governance to try to tackle the collective challenges lying ahead: transparent assessment and remuneration of milk quality, frequent negotiations between the stakeholders (numerous smallholder farmers, milk collection co-operatives and dairy processors) on the sensitive issue of raw milk price, etc. Given the rising volatility of feed and food prices in global markets, the promotion of autonomous dairy chains will be required. That may also be achieved through more regional integration of the national dairy chains between North African countries, by enhanced exchanges of expertise and the encouragement of dairy products’ trade. To realize such a goal, a necessary political will be required, to overcome to current cost of “non Maghreb” - almost 5% of GDP growth according to many economy experts of this region -, which already hampers the effective economic growth of the whole region. That might be a necessary condition to favor food security, promote dairy products’ consumption and avoid risks of social unrest.

## Authors’ original submitted files for images

Below are the links to the authors’ original submitted files for images. Authors’ original file for figure 1 Authors’ original file for figure 2 Authors’ original file for figure 3 Authors’ original file for figure 4
