# Supplementary figures and images for: The dairy chains in North Africa (Algeria, Morocco and Tunisia): from self sufficiency options to food dependency?
Source: Springerplus. 2013 Apr 16;2(1):162. doi: 10.1186/2193-1801-2-162 (PMC3647101; doi:10.1186/2193-1801-2-162)

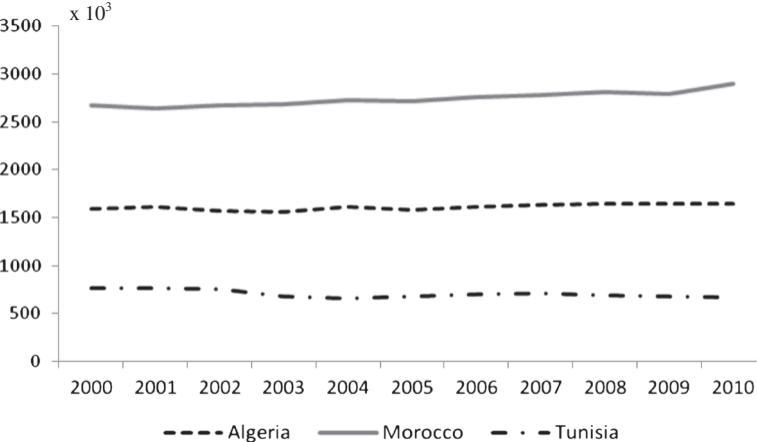

Supplement: Supplementary file 1 — Authors’ original file for figure 1 [file 40064_2012_233_MOESM1_ESM.pdf]

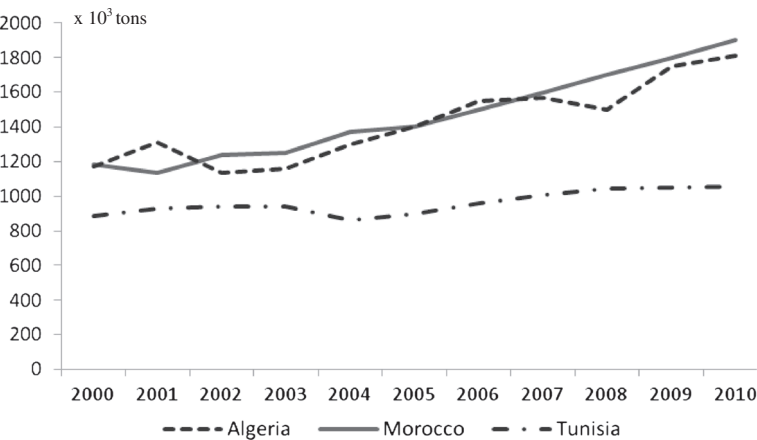

Supplement: Supplementary file 2 — Authors’ original file for figure 2 [file 40064_2012_233_MOESM2_ESM.pdf]

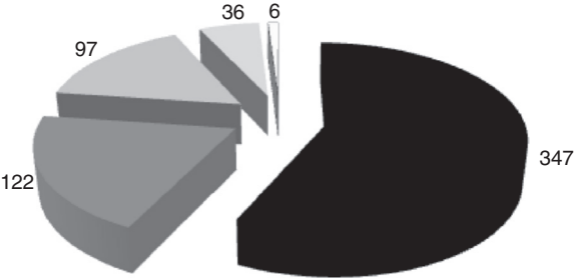

■ Liquid milk ■ Yogurt ■ Cheese ■ Butter □ Milk powder

Supplement: Supplementary file 3 — Authors’ original file for figure 3 [file 40064_2012_233_MOESM3_ESM.pdf]

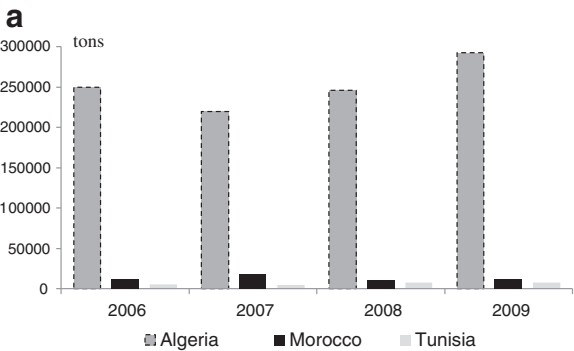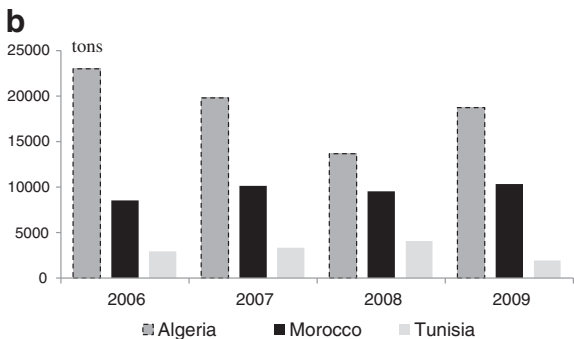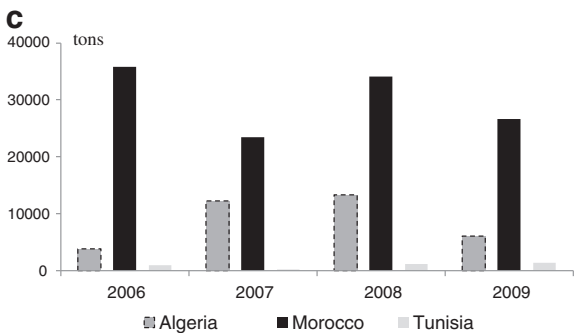

Supplement: Supplementary file 4 — Authors’ original file for figure 4 [file 40064_2012_233_MOESM4_ESM.pdf]
